# From screening-driven medicine to symptom-driven medicine: Da medicina guiada por triagem para a medicina guiada por sintomas

**DOI:** 10.1590/1516-3180.2016.1345290816

**Published:** 2016-09-26

**Authors:** Paulo Andrade Lotufo

**Affiliations:** I MD, DrPH. Full Professor, Department of Internal Medicine, Faculdade de Medicina da Universidade de São Paulo (FMUSP), São Paulo, SP, Brazil.

Each year, there are celebrations of the breast cancer and prostate cancer awareness months, respectively during October and November. In addition to being fundraising movements, these very well-orchestrated worldwide movements have blurred any public health planning for prevention and treatment of other diseases and conditions, except for AIDS. Cancer and AIDS activists do not have limits on obtaining more money and funds. They are the materialization, within medical and public affairs in the 2010s, of the European trade unionist ideology of the second half of the 19^th^ century and the first quarter of the 20^th^ century, when a refrain of "more, more and more" was celebrated.^1^ One undisputable fact is that the propaganda has been successfully reaching lay people. For example, a survey conducted in São Paulo revealed that the population investigated considered that cancer and AIDS were the most important causes of deaths.^2^ However, perusal of the files of the official health statistics for Brazil in 2014 shows that this is not true. In fact, the risk of premature mortality (< 70 years of age) due to breast cancer is almost a quarter of the risk due to stroke; and the rates of prostate cancer are a fifth of those of heart disease.^*^ ^3^

If, on the one hand, the medical-industrial complex relating to cardiovascular diseases has enough power to equilibrate this dispute, on the other hand, health conditions with little or no support exist. These conditions relate to the burden of morbidity with low lethality rates.

The current issue of the Journal presents original articles addressing low-back pain,^3^^,^^4^ frailty,^5^^,^^6^^,^^7^ ankylosis spondylitis^8^ and knee osteoarthritis.^9^ The constant decline in age-adjusted mortality rates for all causes including chronic diseases, combined with the increasing size of the elderly population, is bringing up a new agenda for medical and public health research.^10^ This agenda relates not only to avoidance of lethal diseases, but also to reduction of discomfort and painful conditions.^11^

Current demographic and epidemiological profiles are demanding greater focus on research on the epidemiology of conditions such as low-back and neck pain, frailty, osteoarthritis, migraine, hearing loss, refractive and accommodation errors of vision, depression and anxiety. These conditions are not unique to Brazil, and they are among the top ten leading causes of years lived with disability, according to the Global Burden of Diseases, 2013.^11^

Table 1 shows the top ten conditions that cause years lived with disability (YLD) globally, in developed and developing countries and in Brazil. In decreasing order, the top ten significant illnesses associated with years lived with disability in Brazil are low-back pain, major depressive disorder, anxiety, diabetes, hearing loss, other musculoskeletal conditions, asthma, neck pain, migraine and chronic pulmonary obstructive disease.^11^

**Table 1:**
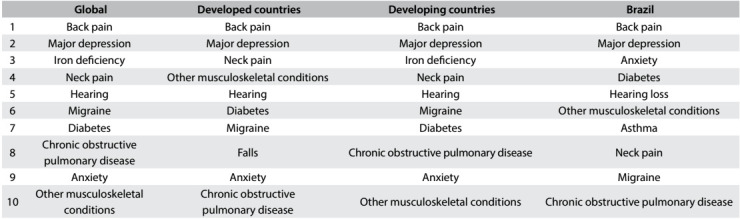
The top ten causes of years lived with disability (YLD) according to the Global Burden of Diseases, 2013

One condition that deserves particular comment is low-back pain. The 2013 Brazilian National Health Survey investigated people over 18 years of age and found that 18.5% of the interviewees reported having some type of complaint relating to the lumbar column. The frequency was higher in urban areas than in rural areas, among women and among people with lower education, and it was age-related, with a plateau at around 27% after 60 years of age.^12^^,^^13^ Although the magnitude of lumbar pain is extremely relevant, the quality of the studies conducted so far has been insufficient, such that they lack internal and external validity to support preventive measures.^14^

Clinical care for osteoarticular complaints, psychiatric diseases, migraine and respiratory disorders needs to have greater presence on the agenda relating to public health. These are conditions that deserve more attention with regard to identifying risk factors and testing new therapies to relieve symptoms. Unfortunately, we are wasting time and money during the breast and prostate cancer awareness months.

## Footnotes

* Available from: http://www.d\atasus.gov.br.
